# Classification of Marine Mammals Using the Trained Multilayer Perceptron Neural Network with the Whale Algorithm Developed with the Fuzzy System

**DOI:** 10.1155/2022/3216400

**Published:** 2022-10-18

**Authors:** Ali Hosseini Nejad Takhti, Abbas Saffari, Diego Martín, Mohammad Khishe, Mokhtar Mohammadi

**Affiliations:** ^1^Department of Information Technology, College of Engineering and Computer Science, Sari Branch, Islamic Azad University, Sari, Iran; ^2^Department of Electrical Engineering, Imam Khomeini Marine Science University, Nowshahr, Iran; ^3^ETSI Telecomunicación, Universidad Politécnica de Madrid, Av. Complutense 30, Madrid 28040, Spain; ^4^Department of Electrical Engineering, Imam Khomeini Marine Science University, Nowshahr, Iran; ^5^Department of Information Technology, College of Engineering and Computer Science, Lebanese French University, Kurdistan Region, Iraq

## Abstract

The existence of various sounds from different natural and unnatural sources in the deep sea has caused the classification and identification of marine mammals intending to identify different endangered species to become one of the topics of interest for researchers and activist fields. In this paper, first, an experimental data set was created using a designed scenario. The whale optimization algorithm (WOA) is then used to train the multilayer perceptron neural network (MLP-NN). However, due to the large size of the data, the algorithm has not determined a clear boundary between the exploration and extraction phases. Next, to support this shortcoming, the fuzzy inference is used as a new approach to developing and upgrading WOA called FWOA. Fuzzy inference by setting FWOA control parameters can well define the boundary between the two phases of exploration and extraction. To measure the performance of the designed categorizer, in addition to using it to categorize benchmark datasets, five benchmarking algorithms CVOA, WOA, ChOA, BWO, and PGO were also used for MLPNN training. The measured criteria are concurrency speed, ability to avoid local optimization, and the classification rate. The simulation results on the obtained data set showed that, respectively, the classification rate in MLPFWOA, MLP-CVOA, MLP-WOA, MLP-ChOA, MLP-BWO, and MLP-PGO classifiers is equal to 94.98, 92.80, 91.34, 90.24, 89.04, and 88.10. As a result, MLP-FWOA performed better than other algorithms.

## 1. Introduction

The deep oceans make up 95% of the oceans' volume, which is the largest habitat on Earth[1]. Creatures are continually being explored in the depths of the ocean with new ways of life [2, 3]. Much research has been conducted in the depths of the ocean, but unfortunately, this research is not enough, and many hidden secrets in the ocean remain unknown [4].

Various species of marine mammals, including whales and dolphins, live in the ocean. Underwater audio signal processing is the newest way to measure the presence, abundance, and migratory marine mammal patterns [5–7]. The use of human-based methods and intelligent methods is one method of recognizing whales and dolphins [8]. Initially, human operator-based methods were used to identify whales and dolphins. Its advantages include simplicity and ease of work. However, the main disadvantage is the dependence on the operator's psychological state and inefficiency in environments where the signal-to-noise ratio is low [9].

To eliminate these defects, automatic target recognition (ATR) based on soft calculations is used [10, 11]. Then, contour-based recognition was used for recognition of whales and dolphins due to its time complexity and low identification rate [12, 13, 14]. The next item from the subset of intelligent methods is the ATR method based on soft computing [15], which is wildly popular due to its versatility and parallel structure [16, 17].

The MLP-NN neural network, due to its simple structure, high performance, and low computational complexity, has become a useful tool for automatically recognizing targets [18–20]. In the past, MLP-NN training used gradient-based methods and error propagation, but these algorithms had a low speed of convergence and were stuck in local minima [21–23].

Therefore, this paper presents a new hybrid method of MLP-NN training using FWOA to classify marine mammals. The main contributions of this work are as follows:

- Practical test design to obtain a real data set from the sound produced by dolphins and whales
- Classifier design using MLP-NN to classify dolphins and whales
- MLP-NN training using the proposed FWOA hybrid technique
- MLP-NN training using new metaheuristic algorithms (CVOA, ChOA, BWO, PGO) and WOA as benchmark algorithms

In the following paragraphs, the paper is structured in such a manner that Section 2 designs an experiment for data collection.  Section 3 will cover how to extract a feature.  Section 4 describes WOA and how to fuzzy.  Section 5 will simulate and discuss it, and finally, Section 6 will conduct conclusions and recommendations.

### 1.1. Background and Related Work

MLP-NNs are a commonly used technology in the field of soft computing [11, 24]. These networks may be used to address nonlinear issues. Learning is a fundamental component of all neural networks and is classified as unsupervised and supervised learning. In most cases, back-propagation techniques or standard [25, 26] are also used as a supervised learning approach for MLP-NNs. Back-propagation is a gradient-based technique with limitations such as slower convergence, making it unsuitable for real world applications. The primary objective of the neural network learning mechanism is to discover the optimal weighted edge and bias combination that produces the fewest errors in network training and test samples [27, 28]. Nevertheless, the majority of MLP-NN faults will remain high for an extended period of time throughout the learning process, after which they will be reduced by the learning algorithm. This is particularly prevalent in mechanisms relying on gradients, such as back-propagation algorithms. In addition, the back-propagation algorithm's convergence is highly dependent on the initial values of the learning rate and the magnitude of the motion. Incorrect values for these variables may potentially result in algorithm diverging. Numerous research works have been conducted to address this issue using the back-propagation algorithm [29], but there is not enough optimization that has occurred, and each solution has unintended consequences. We have seen an increase in the usage of meta-heuristic methods for neural network training in recent years. The following (Table 1) discusses many works on neural network training using different meta-heuristic techniques.

GA and SA are likely to minimize local optimization but have a slower convergence rate. This is inefficient in real-time processing applications. Although PSO is quicker than evolutionary algorithms, it often cannot compensate for poor solution quality by increasing the number of iterations. PSOGSA is a fairly sophisticated algorithm, and its performance is insufficient for solving problems with a high dimension. BBO requires lengthy computations. Despite their minimal complexity and rapid convergence speed, GWO, SCA, and IWT fall victim to local optimization and so are not appropriate for applications requiring global optimization. The primary cause for being stuck in local optimizations is a mismatch between the exploration and extraction stages. Various methods are provided to solve problems such as getting stuck in local optimizations and slow convergence speed in WOA, including parameterization ɑ by the linear control strategy (LCS) and arcsine-based nonlinear control strategy (NCS-arcsine) to establish the right balance between exploration and extraction [51, 52]. LCS and NCS-arcsine strategies usually do not provide appropriate solutions when used for high-dimensional problems.

On the other hand, the no free launch (NFL) theorem logically states that meta-heuristic algorithms do not have the same answer in dealing with different problems [29]. Due to the problems mentioned and considering the NFL theory in this article, a fuzzy whale algorithm called Fuzzy-WOA is introduced for the MLP-NN training problem to identify whales and dolphins.

To investigate the performance of the FWOA, we design an underwater data accusation scenario, create an experimental dataset, and compare it to a well-known benchmark dataset (Watkins et al. 1992). To address the time-varying multipath and fluctuating channel effects, a unique two cepstrum liftering feature extraction technique is used.

## 2. The Experiment Design and Data Acquisition

**Figure 1:**
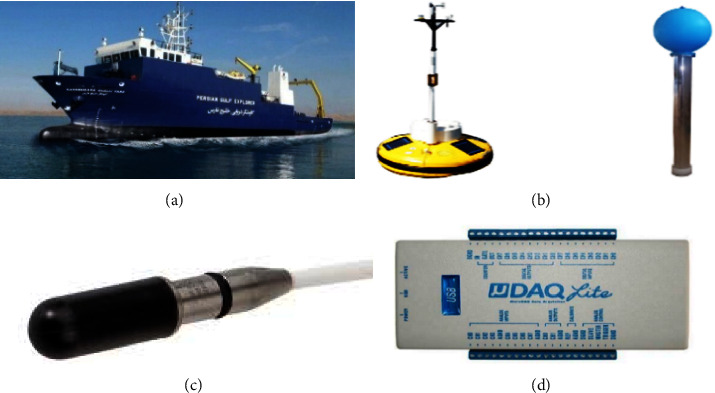
Items needed to collect data sets in: (a) Persian gulf explorer. (b) Research sonobuoys. (c) Hydrophone model 8103 of B&K company. (d) UDAQ_Lite data collection board.

**Figure 2:**
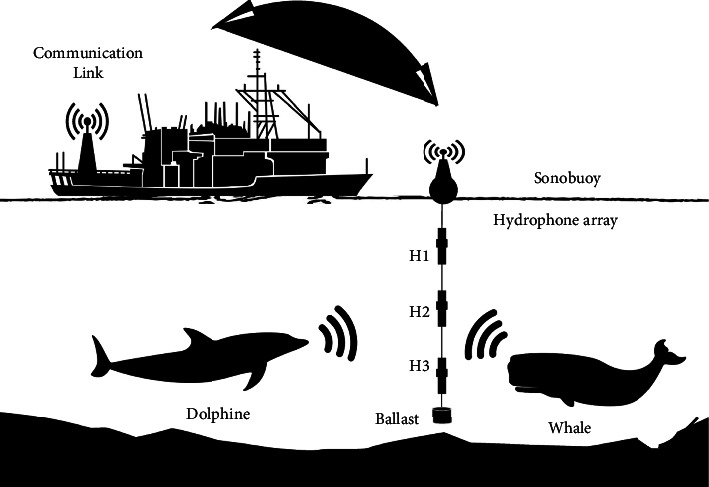
Test scenario and location of the hydrophones.

As shown in Figure 1, to obtain a real data set of sound produced by dolphins and whales from a research ship called the Persian Gulf Explorer and a Sonobuoy, a UDAQ_Lite data acquisition board and three hydrophones (Model B& k 8013) were obtained and were used with equal distance to increase the dynamic range. This test was performed in Bushehr port. The array's length is selected based on the water depth, and Figure 2 shows the hydrophones' location.

The raw data included 170 samples of pantropical spotted dolphin (8 sightings), 180 samples of spinner dolphin (five sightings), 180 cases of striped dolphin (eight sightings), 105 cases of humpback whale (seven sightings), 95 samples of minke whale (five sightings), and 120 samples of the sperm whale (four sightings). The experiment was developed and performed in the manner shown in Figure 2.

### 2.1. The Ambient Noise Reduction and Reverberation Suppression

For example, the sounds emitted by marine mammals (dolphins and whales) recorded by the hydrophone array are considered *x* (*t*), *y* (*t*), *z* (*t*), and the original sound of dolphins and whales is considered *s* (*t*). The mathematical model of the output of hydrophones is in

(1) $$\begin{array}{c} x\left(t\right)=\int_{-\infty }^{t}h\left(t-\tau \right)s\left(\tau \right)\text{,}\\ y\left(t\right)=\int_{-\infty }^{t}g\left(t-\tau \right)s\left(\tau \right)\text{,}\\ z\left(t\right)=\int_{-\infty }^{t}q\left(t-\tau \right)s\left(\tau \right)\text{.} \end{array}$$

In equation (1), the Environment Response Functions (ERF) are denoted by *h* (*t*), *g* (*t*), and *q* (*t*). ERFs are not known, and “tail” is considered uncorrelated [53], and naturally, the first frame of sound produced by marine mammals does not reach the hydrophone array at one time. Due to the sound pressure level (SPL) in the Hydrophone B&K 8103 and reference, which deals with the underwater audio standard, the recorded sounds must be preamplified by a factor of 10^6^.

The frequency domain SPL is then transformed using the Hamming window and fast Fourier transform (FFT). Following that, (2) reduces the frequency bandwidth to 1 Hz.

(2) $$\begin{array}{c} {SPL}_{1}={SPL}_{m}-10\;\log\Delta f\text{.} \end{array}$$

SPLm is the obtained SPL at each fundamental frequency center in dB; re 1 *μ*Pa, SPL1 is the SPL reduced to 1 Hz bandwidth in dB; re 1 *μ*Pa, and Δ*f* represents the bandwidth for each 1/3 Octave band filter. To reduce the square mean error (MSE) between ambient noise and marine mammal noise, a Wiener filter was utilized [54]. Following that, the results were computed using (3) to identify sounds with a low SNR, less than 3 dB, that should be eliminated from the database.

(3) $$\begin{array}{c} {SPL}_{v}=10\log\left[10^{\left(\frac{S{PL}_{T}}{10}\right)}-10^{\left(\frac{S{PL}_{A}}{10}\right)}\right]\text{,} \end{array}$$

where *T*, *V*, and *A* represent all the available signals, sound, and ambient sound, respectively. After that, the SPLs were recalculating at a standard measuring distance of 1 m as follows:

(4) $$\begin{array}{c} SPL=SPL1+20\log\left(r\right)\text{.} \end{array}$$

**Figure 3:**
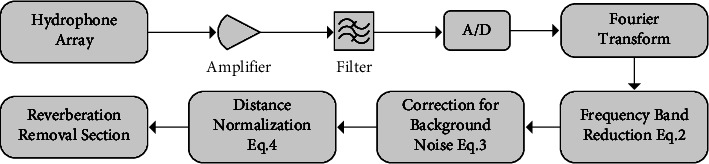
The block diagram of the ambient noise reduction and reverberation suppression system.

Figure 3 illustrates the block diagram for ambient noise reduction and reverberation suppression.

**Figure 4:**
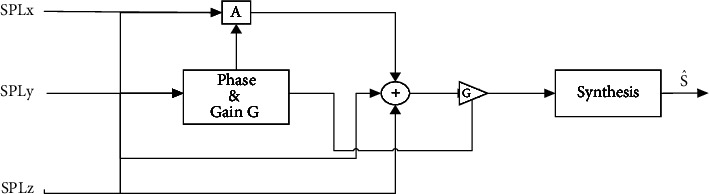
The reverberation removal section's block diagram.

**Figure 5:**
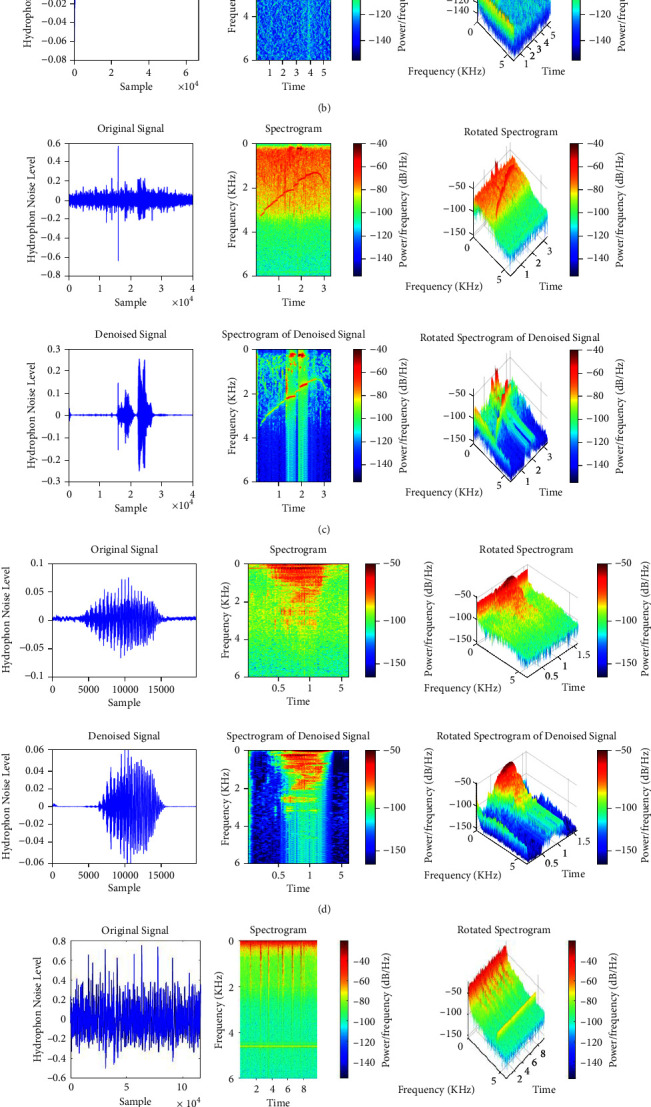
Typical sound presentations produced by dolphins and whales and their spectrogram. (a) Pantropical spotted dolphin. (b) Spinner dolphin. (c) Striped dolphin. (d) Humpback whale. (e) Minke whale. (f) Sperm whale.

In the next part, the effect of reverberation must be removed. In this regard, the common phase is added to the band (reducing the phase change process using the delay between the cohesive parts or the initial sound is called the common phase) [55]. Therefore, a cross-correlation pass function by adjusting each frequency band's gain eliminates noncorrelated signals and passes the correlated signals. Finally, the output signals from each frequency band are merged to form the estimated signal, i. e., $\hat{S}$. The basic design for removing reverberation is shown in Figure 4.  Figure 5 illustrates typical representations of dolphin and whale sounds and melodies, as well as their spectra.

## 3. Average Cepstral Features and Cepstral Liftering Features

The effect of ambient noise and reverberation decreases after detecting the audio signal frames obtained in the preprocessing stage. In the next step, the detected signal frames enter the feature extraction stage. The sounds made by dolphins and whales emitted from a distance to the hydrophone experience changes in size, phase, and density. Due to the time-varying multipath phenomenon, fluctuating channels complicate the challenge of recognizing dolphins and whales. The cepstral factors combined with the cepstral liftering feature may considerably reduce the impacts of multipath, whilst the average cepstral coefficients can significantly minimize the time-varying effects of shallow underwater channels [56]. As a result, this section recommends the use of cepstral features, such as mean cepstral features and cepstral liftering features, in order to construct a suitable data set. Cepstrum indices of greater and lower values indicate that the channel response cepstrum and the original sound of dolphins and whale cepstrum are distinct. [57]. They are located in distinct regions of the liftering cepstrum. Therefore, by reducing time liftering, the quality of the features is increased. Following removing noise and reverberation, the frequency domain frames of SPLs (S (k)) are passed to the portion extracting cepstrum features. The following equation determines the cepstrum characteristics of the sound produced by dolphin and whale signals.

(5) $$\begin{array}{c} c\left(n\right)=\overset{M}{\underset{l=1}{\sum }}\log\left(\overset{N-1}{\underset{k=0}{\sum }}{\left|S\left(k\right)\right|}^{2}H_{l}\left(k\right)\right)\cos\left(n/\left(1-0.5\right)\pi /M\right)\text{,} \end{array}$$

where *S*(*k*) indicates the frequency domain frames of sounds generated by dolphins and whales, *N* signifies the number of discrete frequencies employed in the FFT, and Hl (*k*) denotes the transfer function of the Mel-scaled triangular filter with *l* = 0,1, ..., *M*. Ultimately, using the discrete cosine transform (DCT), the cepstral coefficients are converted to the time domain as *c* (*n*).

As previously stated, the sound generated by dolphins and whales is obtained via a method called low-time liftering. Consequently, (6) is recommended to separate the sound that originated from the whole sound.

(6) $$\begin{array}{c} w_{e}\left[n\right]=\left\{\begin{array}{cc} 1\text{,} & 0\leq n\leq L_{c}\text{,}\\ 0\text{,} & L_{c}\leq n\leq \frac{N}{2}\text{.} \end{array}\right. \end{array}$$

*L*_c_ indicates the liftering window's length, which is typically 15 or 20. The ultimate features may be computed by multiplying the cepstrum *c* (*n*) by and using the logarithm and DFT functions as described in the following equations:

(7) $$\begin{array}{c} c_{e}\left(n\right)=w_{e}\left[n\right]c\left(n\right)\text{,} \end{array}$$

(8) $$\begin{array}{c} c_{L}\left(n\right)=\mathrm{Log}\left(DFT\left(c_{e}\left(n\right)\right)\right)\text{.} \end{array}$$

Finally, the feature vector would be represented using

(9) $$\begin{array}{c} x_{m}=\left[c_{L}\left(0\right),c_{L}\left(1\right),\cdots ,{c_{L}\left(P-1\right)}^{T}\right]\text{.} \end{array}$$

The first 512-cepstrum points (out of 8192 points in one frame for a sampling rate of 8192 Hz, expect for zeroth index {*c*_*y*_^*m*^[0]} are corresponded to 62.5 ms liftering coefficients and are windowed from the *N* indices, which is equivalent to one frame length to reduce the liftering coefficients to 32 features. Prior to averaging, the duration of subframes is five seconds. Ten prior frames compose 50 s average cepstral features throughout the averaging liftering technique, smoothing 10 frame results in the final average cepstral features. As a result, the average cepstral feature vector has 32 elements. The *X*_*m*_ vector would then be used as an input signal for an MLP-NN in the subsequent phase.

**Figure 6:**
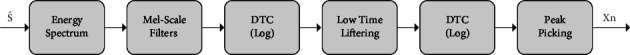
The procedure of extracting cepstrum liftering features.

**Figure 7:**
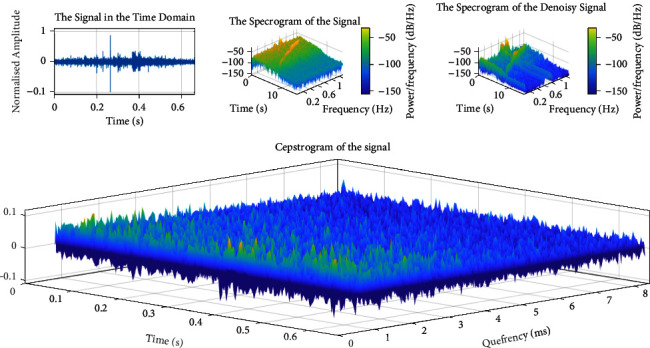
The typical visualization produces sound cepstrum features (striped dolphin).

To summarize, the number of inputs to a neural network equals P. The whole feature extraction step is shown in Figure 6. To summarize, Figure 7 depicts the result of this step.

## 4. Design of an FWOA-MLPNN for Automatic Detection of Sound Produced by Marine Mammals

MLP-NN is the simplest and most widely used neural network [58, 59]. Important applications of MLP-NN include automatic target recognition systems. For this reason, this article uses MLP-NN as an identifier [60]. MLP-NN is amongst the most durable neural networks available and is often used to model systems with a high degree of nonlinearity. In addition, the MLP-NN is a feed-forward network able of doing more precise nonlinear fits. Despite what has been said, one of the challenges facing MLP-NN is always training and adjusting the edges' bias and weight [61].

**Figure 8:**
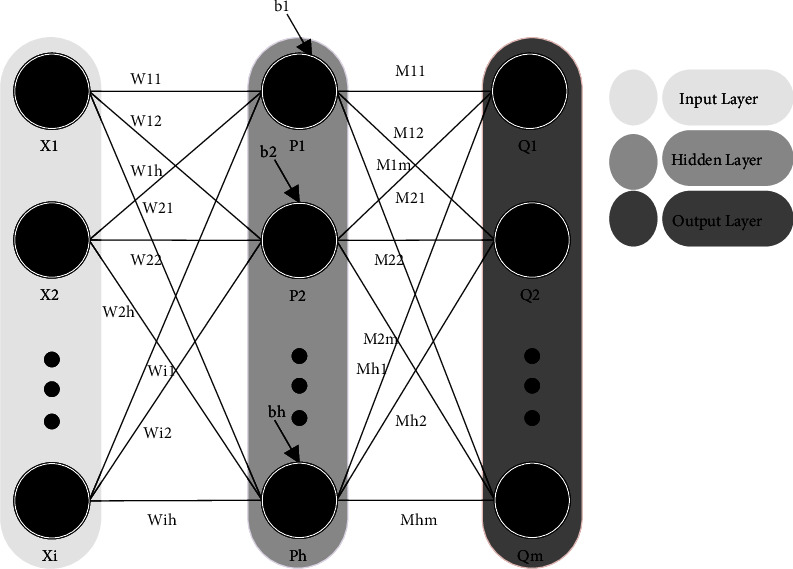
MLP-NN as a search agent for a meta-heuristic method.

The steps for using meta-heuristic algorithms to teach MLPNN are as follows: the first phase is to determine how to display the connection weights. The second phase involves evaluating the fitness function in order to determine the connection weights, which may be thought of as the mean square error (MSE) for recognition issues. The third step employs the evolutionary process to minimize the fitness function, that is represented by the MSE.  Figure 8 and equation (10) illustrate the technical design of the evolutionary technique for connection weight training.

(10) $$\begin{array}{c} \overset{⟶}{V}=\left\{\overset{⟶}{W}.\overset{⟶}{\theta }\right\}=\left\{W_{11}.W_{12}.\cdots .W_{ih}.b_{1}.b_{2}.\cdots .b_{h}.M_{11}.M_{12}.\cdots .M_{hm}\right\}\text{,} \end{array}$$

where *n* represents the input nodes' amount, *W*_*ij*_ indicating the node's connection weight to the *j*^*th*^ node, *θ*_*j*_denotes the bias (threshold) of the *j*^*th*^ hidden neuron.

As noted before, the MSE is a frequently used criterion for assessing MLP-NNs, as the following equation demonstrates.

(11) $$\begin{array}{c} MSE=\overset{m}{\underset{i=1}{\sum }}{\left(o_{i}^{k}-d_{i}^{k}\right)}^{2}\text{,} \end{array}$$

where *m* is the number of neurons in the MLP outputs, *d*_*i*_^*k*^ is the optimal output of the *i*^*th*^ input unit in cases where *k*^*th*^ training sample is utilized, and *o*_*i*_^*k*^ denotes the real output of the *i*^*th*^ input unit in cases where the *k*^*th*^ training sample is observed in the input. To be successful, the MLP must be tuned to a collection of training samples. As a result, MLP performance is calculated as follows:

(12) $$\begin{array}{c} E=\overset{T}{\underset{k=1}{\sum }}\overset{m}{\underset{i=1}{\sum }}{\left(o_{i}^{k}-d_{i}^{k}\right)}^{2}/T\text{.} \end{array}$$

T denotes the number of training samples, *d*_*i*_^*k*^ denotes the optimal output related to i^th^ input when using *k*^th^ the training sample, *m* denotes the number of outputs, and *O*_*i*_^*k*^indicates the input's real output when using *k*^*th*^ the training sample. Finally, the recognition system requires a meta-heuristic method to fine-tune the parameters indicated above. The next subsection proposes an instructor based on an improved whale optimization algorithm (WOA) with fuzzy logic called FWOA.

### 4.1. Fuzzy WOA

This section upgrades WOA using fuzzy inference. In this regard, in the first subsection, it will review WOA, and in the second subsection, it will describe the fuzzy method for upgrading WOA.

#### 4.1.1. Whale Optimization Algorithm

The WOA optimization algorithm was introduced in 2016, inspired by the way whales were hunted by Mirjalili and Lewis [62]. WOA starts with a set of random solutions. In each iteration, the search agents update their position by using three operators: encircling prey, bubble-net attack (extraction phase), and bait search (exploration phase). In encircling prey, whales detect prey and encircle it. The WOA assumes that the best solution right now is his prey. Once the optimal search agent is discovered, all other search agents will update their position to that of the optimal search agent. The following equations express this behavior:

(13) $$\begin{array}{c} \overset{⟶}{D}=\left|\overset{⟶}{C}∙\overset{⟶}{X^{*}}\left(t\right)-\overset{⟶}{X}\left(t\right)\right|\text{,} \end{array}$$

(14) $$\begin{array}{c} \overset{⟶}{X}\left(t+1\right)=\overset{⟶}{X^{*}}\left(t\right)-\overset{⟶}{A}∙\overset{⟶}{D}\text{,} \end{array}$$

where *t* is the current iteration, $\overset{⟶}{A}$ and $\overset{⟶}{C}$ are the coefficient vectors, $\left(\overset{⟶}{X^{*}}\right)$ is the place vector which is the best solution so far, and $\overset{⟶}{X}$ is the place vector. It should be noted that in each iteration of the algorithm, if there is a better answer, $\left(\overset{⟶}{X^{*}}\right)$ should be updated. The vectors $\overset{⟶}{A}$ and $\overset{⟶}{C}$ are obtained using the following equations:

(15) $$\begin{array}{c} \overset{⟶}{A}=2\overset{⟶}{\alpha }∙\overset{⟶}{r}-\overset{⟶}{\alpha }\text{,} \end{array}$$

(16) $$\begin{array}{c} \overset{⟶}{C}=2∙\overset{⟶}{r}\text{,} \end{array}$$

where $\overset{⟶}{\alpha }$ decreases linearly from 2 to zero during repetitions and $\overset{⟶}{r}$ is a random vector in the distance [0, 1]. During the bubble-net assault, the whale swims simultaneously around its victim and along a contraction circle in a spiral pattern. To describe this concurrent behavior, it is anticipated that the whale would adjust its location during optimization using either the contractile siege mechanism or the spiral model. (17) is the mathematical model for this phase.

(17) $$\begin{array}{c} \overset{⟶}{X}\left(t+1\right)=\left\{\begin{array}{cc} \overset{⟶}{X^{*}}\left(t\right)-\overset{⟶}{A}∙\overset{⟶}{D}\text{,} & ifp<0.5\text{,}\\ \overset{⟶}{D}∙e^{bi}∙\cos\left(2\pi l\right)\text{,} & ifp\geq 0.5\text{,} \end{array}\right. \end{array}$$

where $\overset{⟶}{D}$ is derived from (8) and denotes the distance i between the whale and its prey (the best solution ever obtained). A constant *b* is used to define the logarithmic helix shape; *l* is a random number between −1 and +1. *p* is a random number between zero and one. Vector A is used with random values between −1 and 1 to bring search agents closer to the reference whale. In the search for prey to update the search agent's position, random agent selection is used instead of using the best search agent's data. The mathematical model is in the form of the following equations:

(18) $$\begin{array}{c} \overset{⟶}{D}=\left|\overset{⟶}{C}\right|∙\overset{⟶}{X_{rand}}∙\overset{⟶}{X}\text{,} \end{array}$$

(19) $$\begin{array}{c} \overset{⟶}{X}\left(t+1\right)=\overset{⟶}{X_{rand}}-\overset{⟶}{A}∙\overset{⟶}{D}\text{.} \end{array}$$

**Figure 9:**
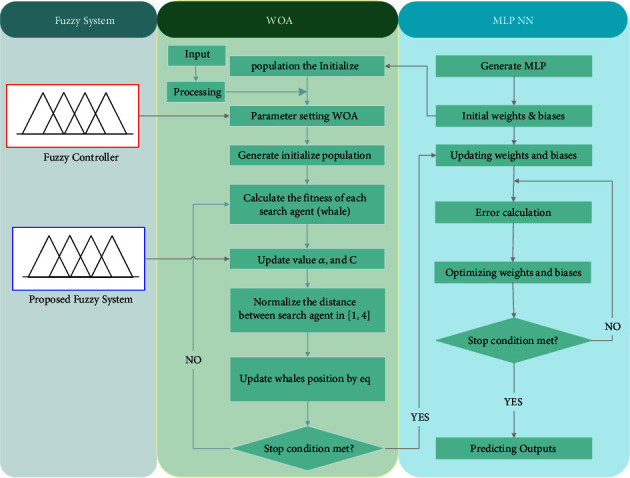
Flowchart of the WOA.

**Figure 10:**
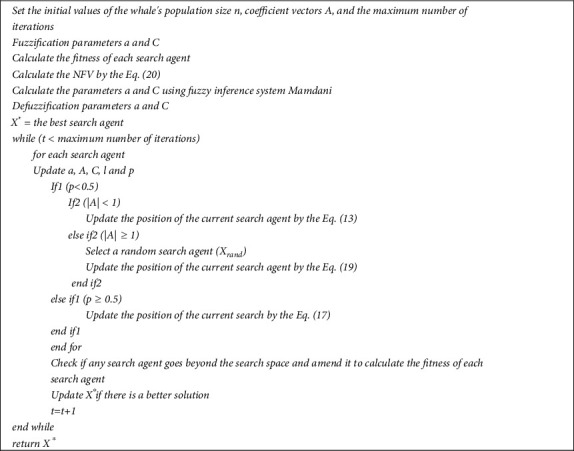
Pseudocode of the FWOA.

$\overset{⟶}{X_{rand}}$ is the randomly chosen position vector (random whale) for the current population, and vector $\overset{⟶}{A}$ is utilized with random values larger or smaller to one to drive the search agent away from the reference whale.  Figure 9 shows the FWOA flowchart, and Figure 10 shows the pseudocode of the FWOA. In the next section, we will describe the proposed fuzzy system.

#### 4.1.2. Proposed Fuzzy System for Tuning Control Parameters

The proposed fuzzy model receives the normalized performance of each whale in the population (normalized fitness value) and the current values of the parameters $\overset{⟶}{\alpha }$ and $\overset{⟶}{C}$. The output also shows the amount of change using the symbols ∆*α* and ∆*C*. The NFV value for each whale is obtained as follows:

(20) $$\begin{array}{c} NFV=\frac{fitness-{fitness}_{\min}}{{fitness}_{\min}-{fitness}_{\max}}\text{.} \end{array}$$

The NFV value is in the range of [0. 1]. This paper's optimization problem is of the minimization type, in which the fitness of each whale is obtained directly by the optimal amount of these functions. (21) and (22) update the parameters $\overset{⟶}{\alpha }$ and $\overset{⟶}{C}$ for each whale which are as follows:

(21) $$\begin{array}{c} {\overset{⟶}{\alpha }}^{t+1}={\overset{⟶}{\alpha }}^{t}+∆\alpha \text{,} \end{array}$$

(22) $$\begin{array}{c} {\overset{⟶}{C}}^{t+1}={\overset{⟶}{C}}^{t}+∆C\text{.} \end{array}$$

**Figure 11:**
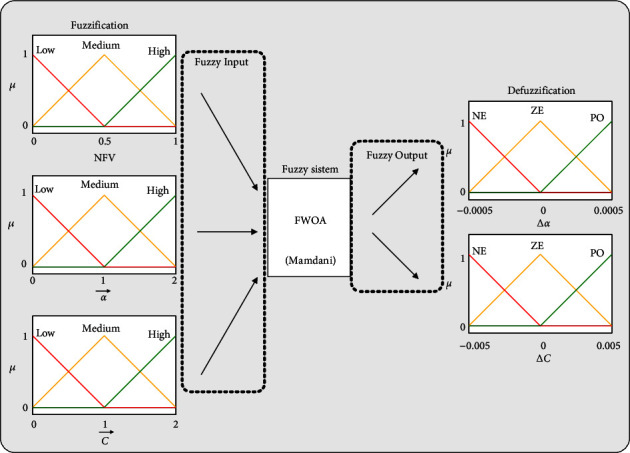
A proposed fuzzy model for setting parameters $\overset{⟶}{\alpha }$ and $\overset{⟶}{C}$.

The fuzzy system is responsible for updating the parameters $\overset{⟶}{\alpha }$ and $\overset{⟶}{C}$ of each member of the population (whale) and the three inputs of this system are the current value of parameters $\overset{⟶}{\alpha }$ and $\overset{⟶}{C}$, NFV. Initially, these values are “fuzzification” by membership functions. Then, their membership value is obtained using *μ*. These values apply to a set of rules and give the values ∆*α* and ∆C. After determining these values, the “defuzzification” process is performed to estimate the numerical values ∆*α* and ∆*C*. Finally, these values are applied in (12) and (13) to update the parameters ∆*α* and ∆*C*. The fuzzy system used in this article is of the Mamdani type.  Figure 11 shows the proposed fuzzy model and membership functions used to adjust the whale algorithm's control parameters. The adjustment range for membership functions is obtained using the primary [62] of the WOA. Many experiments were performed for all types of membership functions, including trimf, trapmf, gbellmf, gaussmf, gauss2mf, sigmf, dsigmf, psigmf, pimf, smf, and zmf. Comparison of the results showed that trimf input and output membership functions are more suitable for using the data set obtained in Sections 2 and 3.

**Figure 12:**
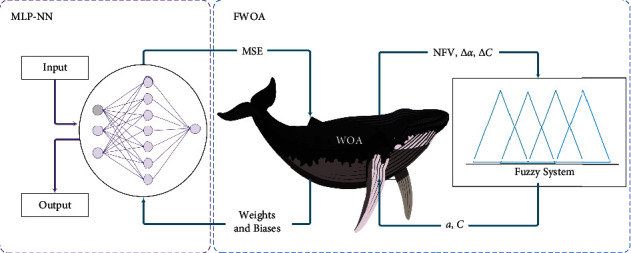
How to train MLP-NN using FWOA.

The semantic values used in the membership functions of the input variables $\overset{⟶}{\alpha }$ , $\overset{⟶}{C}$, and NFV are low, medium, and high. The semantic values used in the output variables ∆*α* and ∆C are NE (negative), ZE (zero), and PO (positive). The fuzzy rules used are presented in Table 2, and how to train MLP-NN using FWOA is shown in Figure 12.

## 5. Simulation Results and Discussion

The evaluation of the IEEE CEC-2017 benchmark functions is presented in this section, followed by a discussion of the results achieved for the classification of marine mammals.

### 5.1. Evaluation of IEEE CEC-2017 Benchmark Functions

The CEC-2017 benchmark functions and dimension size are shown in Table 3.  Table 6 shows the parameters selected in the algorithms used for the benchmark functions. In all algorithms, the maximum number of iterations is 100, and the population size is 180.

As shown in Table 4, the FWOA algorithm has achieved more encouraging results compared to CVOA, WOA, ChOA, BWO, and PGO. From a more detailed comparison of WOA with its upgraded version with a fuzzy subsystem (FWOA), it can be seen that the improvement and upgrade of WOA have been successful.

### 5.2. Classification of Marine Mammals

**Figure 13:**
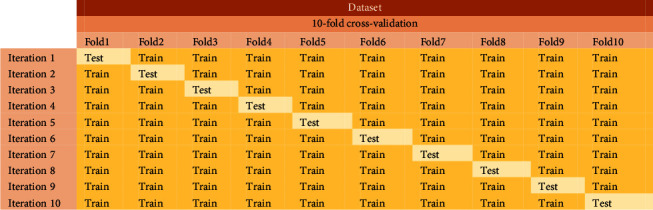
The 10-fold cross-validation.

In this section, to show the power and efficiency of MLP-FWOA, in addition to using the sounds obtained in Sections 2 and 3, the reference dataset (Watkins et al. 1992) is also used. As already mentioned, To obtain the data set, the *X*_*m*_ vector is assumed to be an input for the MLP-WOA. The *X*_*m*_ dimension is 680 × 42, which indicates that there are 42 features and 680 samples in the data set. In addition, the benchmark dataset has a dimension of 410 × 42. In MLP-FWOA, the number of input nodes is equal to the number of features. The 10-fold cross-validation method is used to evaluate the models. Therefore, first, the data are divided into ten parts, and each time nine parts are used for training and another part for testing.  Figure 13 shows the 10-fold cross-validation. The final classification rate for each classifier is calculated using the average of the ten classification rates obtained.

To have a fair comparison between the algorithms, the condition of stopping 300 iterations is considered. There is no specific equation for obtaining the number of hidden layer neurons, so (23) is used to obtain [63].

(23) $$\begin{array}{c} H=2\times N+1\text{,} \end{array}$$

where *N* indicates the total number of inputs and *H* indicates the total number of hidden nodes. Furthermore, the number of output neurons corresponds to the number of marine mammal classifications, namely six.

For a comprehensive assessment of FWOA performance, this algorithm is compared with WOA [62], ChOA [64], PGO [65], CVOA [66], and BWO [67] benchmark algorithms.

In all population base algorithms, population size is a hyper parameter that plays a direct role in the algorithm's performance in the search space. For this reason, many experiments were performed with different population numbers, some of which are shown in Table 5. The results showed that for the proposed model, a population of 180 is the most appropriate value. In other words, with an increasing population, in addition to no significant improvement in model performance, complexity increases, and processing time increases.  Table 6 illustrates the fundamental parameters and major values of various benchmark methods. The classification rate to adjust the population size of different algorithms for the data set is obtained from parts 2 and 3.

The classifiers' performance is then tested for the classification rate, local minimum avoidance, and convergence speed. Each method is run 40 times, and the classification rate, mean, and standard deviation of the smallest error, the A20-index [68], and the *p* value are listed in Tables 7 and 8. The mean and standard deviation of the smallest error, the A20-index, and the *p* value all reflect how well the method avoids local optimization.

**Figure 14:**
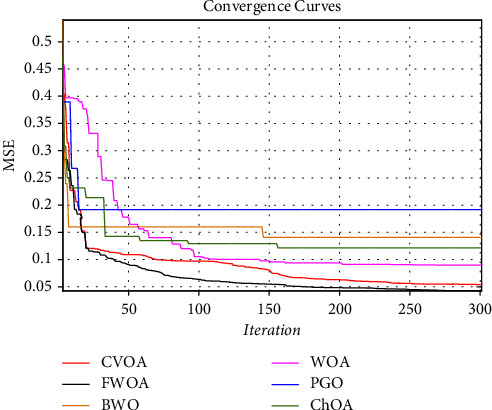
Convergence diagram of different training algorithms for dataset reference [42].

**Figure 15:**
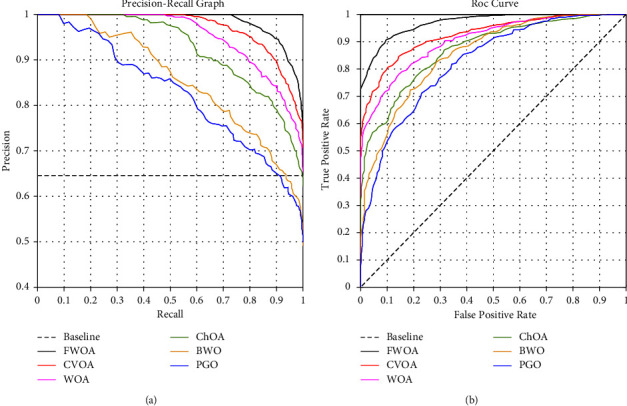
Receiver operating characteristic for dataset reference [42].

**Figure 16:**
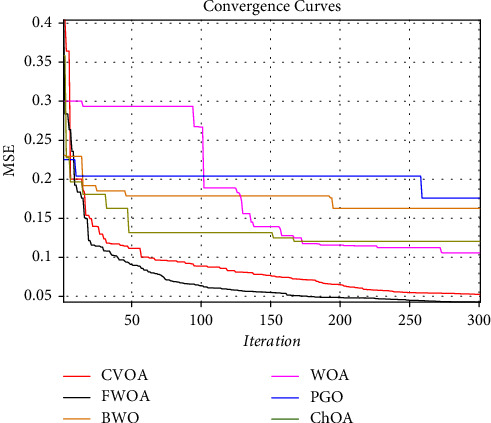
Convergence diagram of different training algorithms for datasets obtained in parts 2 and 3.

**Figure 17:**
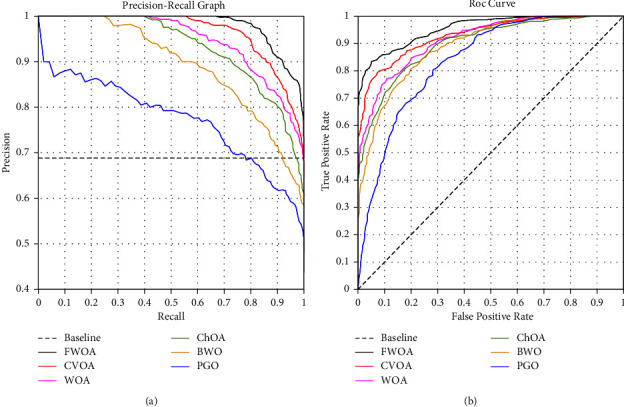
Receiver operating characteristic for datasets obtained in parts 2 and 3.

Figures 14 and 16 show a comprehensive comparison of the convergence speed and syntax and the classifiers' final error rate. Figures 15 and 17 show a receiver operating characteristic for datasets.

The simulation was conducted in MATLAB 2020a by using a personal computer with a 2.3 GHz processor and 5 GB RAM.

As shown in Figures 14 and 16., among the benchmark algorithms used for MLP training, FWOA has the highest convergence speed. PGO has the lowest convergence speed by adjusting control parameters by fuzzy inference, correctly detecting the boundary between the exploration and extraction phases. As shown in Tables 7 and 8, MLP-FWOA has the highest classification rate, and MLP-PGO has the lowest classification rate among the classifiers. The STD values, shown in Tables 7 and 8, indicate that the MLP-FWOA results rank first in the two datasets, confirming that the FWOA performs better than other standard training algorithms and demonstrates the FWOA's ability to avoid getting caught up in local optimism. A *p* value of less than 0.05 indicates a significant difference between FWOA and other algorithms. According to Tables 7 and 8, a20-index is 1 for all predictive classifiers. It confirms that all models can provide good results for similar data as well.

Adding a subsystem to a metaheuristic algorithm increases its complexity. However, a comparison of the convergence curves in Figures 14 and 16 shows that the FWOA achieved the global optimum faster than the other algorithms used. Other algorithms were stuck in the local optimum if they converged. In particular, by comparing the WOA and FWOA in Figures 14 and 16, it can be seen that using an auxiliary (fuzzy system) subsystem is necessary to avoid getting caught up in the local optimum in the WOA. In general, using a fuzzy system to improve WOA increases complexity. However, the convergence curves and better performance of FWOA than other algorithms used show a reduction in computational cost. The reduced MSE of this method compared to other algorithms employed is more indicative that despite increased complexity, FWOA performance is improving.

## 6. Conclusions and Recommendations

In this paper, to classify marine mammals, a fuzzy model of control parameters of the whale optimization algorithm was designed to train an MLP-NN. CVOA, WOA, FWOA, Ch0A, PGO, and BWO algorithms have been used for the MLP-NN training stage. As the simulation results show, FWOA has a powerful performance in identifying the boundary between the exploration and extraction phases. For this reason, it can identify the global optimal and avoid local optimization. The results indicate that MLP-FWOA, MLP-CVOA, MLP-WOA, MLP-ChOA, MLPBWO, and MLP-PGO have better performance for classifying the sound produced by marine mammals. The convergence curve also shows that FWOA converges faster than the other five benchmark algorithms in convergence speed.

Due to the complex environment of the sea and various unwanted signals such as reverberation, clutter, and types of noise in the seabed, lack of access to data sets with a specific signal-to-noise ratio is one of the main limitations of the research.

For future research directions, we recommend the following list of topics:

- MLP-NN training using other metaheuristic algorithms for the classification of marine mammals
- Using other artificial neural networks and using deep learning for the classification of marine mammals
- Direct use of metaheuristic algorithms as classifiers for classification of marine mammals.

## Figures and Tables

**Table 1:** Some related studies.

Paper	Type	Application	Training algorithms	Year
[30]	Feed-forward NN	Sonar image classification	Genetic algorithm (GA)	1989
[31]	MLP NN	Magnetic body detection in a magnetic field	Simulated annealing (SA)	1994
[32]	MLP NN	Predicting the solubility of gases in polymers	Particle swarm optimizer (PSO)	2013
[33]	MLP NN	Parkinson's disease diagnosis	Social-spider optimization (SSA)	2014
[34]	ANN	Artificial intelligence	Runge Kutta method	2015
[35]	MLP NN	XOR, heart, iris, balloon, and breast cancer dataset	Moth-flame optimization (MFO)	2016
[36]	MLP NN	Sonar dataset classification	Gray wolf optimization (GWO)	2016
[37]	Feed-forward NN	Sonar dataset classification	Particle swarm optimizer (PSO)	2017
[38]	Algorithm improvement	23 benchmark functions and solving infinite impulse response model identification	Lévy flight trajectory-based WOA (LWOA)	2017
[39]	MLP NN	Big data	Biogeography-based optimization (BBO)	2018
[40]	MLP NN	UCI dataset (benchmark)	Monarch butterfly optimization (MBO)	2018
[41]	Algorithm improvement	Five standard engineering optimization problems	LWOA	2018
[42]	MLP NN	Sonar target classification	Dragonfly optimization algorithm (DOA)	2019
[42]	MLP NN	Sonar dataset classification	Improved whale	2019
[43]	MLP NN	Classification of EEG signals	PSOGSA	2020
[44]	ANN	Prediction of urban stochastic water demand	Slime mould algorithm (SMA)	2020
[45]	Algorithm improvement	Optimize star sensor calibration	Hybrid WOA-LM algorithm	2020
[46]	Feed-forward NN	COVID19 detection	Colony predation algorithm (CPA)	2020
[47]	MLP NN	XOR, balloon, Iris, breast cancer, and heart	Harris hawks optimization (HHO)	2020
[48]	Deep convolutional NN	COVID19 detection	Sine-cosine (SCA)	2021
[49]	ANN	Predicting ground vibration	Hunger games search optimization (HGS)	2021
[50]	MLP NN	Sonar dataset classification	Fuzzy grasshopper optimization algorithm (FGOA)	2022

**Table 2:** Applied fuzzy rules.

If (NFV is low) and ($\overset{⟶}{\alpha }$ is low), then (∆*α* is ZE)
If (NFV is low) and ($\overset{⟶}{\alpha }$ is medium), then (∆*α* is NE)
If (NFV is low) and ($\overset{⟶}{\alpha }$ is high), then (∆*α* is NE)
If (NFV is medium) and ($\overset{⟶}{\alpha }$ is low), then (∆*α* is PO)
If (NFV is medium) and ($\overset{⟶}{\alpha }$ is medium), then (∆*α* is ZE)
If (NFV is medium) and ($\overset{⟶}{\alpha }$ is high), then (∆*α* is NE)
If (NFV is high) and ($\overset{⟶}{\alpha }$ is low), then (∆*α* is PO)
If (NFV is high) and ($\overset{⟶}{\alpha }$ is medium), then (∆*α* is ZE)
If (NFV is high) and ($\overset{⟶}{\alpha }$ is high), then (∆*α* is NE)
If (NFV is low) and ($\overset{⟶}{C}$ is low), then (∆C is PO)
If (NFV is low) and ($\overset{⟶}{C}$ is medium), then (∆C is PO)
If (NFV is low) and ($\overset{⟶}{C}$ is high), then (∆C is ZE)
If (NFV is medium) and ($\overset{⟶}{C}$ is low), then (∆C is PO)
If (NFV is medium) and ($\overset{⟶}{C}$ is medium), then (∆C is ZE)
If (NFV is medium) and ($\overset{⟶}{C}$ is high), then (∆C is NE)
If (NFV is high) and ($\overset{⟶}{C}$ is low), then (∆C is PO)
If (NFV is high) and ($\overset{⟶}{C}$ is medium), then (∆C is ZE)
If (NFV is high) and ($\overset{⟶}{C}$ is high), then (∆C is NE)

**Table 3:** IEEE CEC-2017 benchmark test functions.

No.	Functions	Dim	*f*_min_
*f*_1_	Shifted and rotated bent cigar function	30	100
*f*_2_	Shifted and rotated sum of different power function	30	200
*f*_3_	Shifted and rotated Zakharov function	30	300
*f*_4_	Shifted and rotated Rosenbrock's function	30	400
*f*_5_	Shifted and rotated Rastrigin's function	30	500
*f*_6_	Shifted and rotated expanded Scaffer's function	30	600
*f*_7_	Shifted and rotated Lunacek Bi_Rastrigin function	30	700
*f*_8_	Shifted and rotated noncontinuous Rastrigin's function	30	800
*f*_9_	Shifted and rotated Lévy function	30	900
*f*_10_	Shifted and rotated Schwefel's function	30	1000
*f*_11_	Hybrid function 1 (*N* = 3)	30	1100
*f*_12_	Hybrid function 2 (*N* = 3)	30	1200
*f*_13_	Hybrid function 3 (*N* = 3)	30	1300
*f*_14_	Hybrid function 4 (*N* = 4)	30	1400
*f*_15_	Hybrid function 5 (*N* = 4)	30	1500
*f*_16_	Hybrid function 6 (*N* = 4)	30	1600
*f*_17_	Hybrid function 6 (*N* = 5)	30	1700
*f*_18_	Hybrid function 6 (*N* = 5)	30	1800
*f*_19_	Hybrid function 6 (*N* = 5)	30	1900
*f*_20_	Hybrid function 6 (*N* = 6)	30	2000
*f*_21_	Composition function 1 (*N* = 3)	30	2100
*f*_22_	Composition function 2 (*N* = 3)	30	2200
*f*_23_	Composition function 3 (*N* = 4)	30	2300

**Table 4:** AVG and STD deviation of the best optimal solution for 40 independent runs on IEEE CEC-2017 benchmark test functions.

FUNC		Algorithm					
		CVOA	WOA	FWOA	ChOA	PGO	BWO
*f*_1_	AVG	1.08*E* + 03	3.41*E* + 11	3.91*E* + 01	4.04*E* + 02	1.24*E* + 11	1.72*E* + 11
	STD	4.48*E* + 04	6.06*E* + 08	3.41*E* + 02	4.34*E* + 02	2.38*E* + 10	2.83*E* + 08

*f*_2_	AVG	3.01*E* + 03	6.51*E* + 08	3.20*E* + 02	9.81*E* + 04	6.76*E* + 08	6.15*E* + 08
	STD	7.81*E* + 04	9.04*E* + 08	9.87*E* + 01	7.77*E* + 05	1.41*E* + 08	2.82*E* + 07

*f*_3_	AVG	2.40*E* + 03	7.07*E* + 03	6.96*E* + 03	2.46*E* + 03	7.08*E* + 03	7.49*E* + 05
	STD	1.21*E* + 03	8.96*E* + 02	9.55*E* + 04	7.71*E* + 02	5.26*E* + 04	1.32*E* + 03

*f*_4_	AVG	4.79*E* + 01	1.47*E* + 03	5.13*E* + 03	5.43*E* + 01	1.70*E* + 04	1.62*E* + 04
	STD	1.38*E* + 02	1.96*E* + 02	9.75*E* + 01	3.38*E* + 02	6.15*E* + 03	2.48*E* + 03

*f*_5_	AVG	6.68*E* + 03	8.91*E* + 01	5.20*E* + 01	5.66*E* + 01	8.12*E* + 03	8.10*E* + 03
	STD	2.68*E* + 02	1.30*E* + 02	1.80*E* + 00	1.10*E* + 02	2.83*E* + 02	2.36*E* + 02

*f*_6_	AVG	6.38*E* + 03	6.78*E* + 03	6.01*E* + 01	6.01*E* + 01	6.78*E* + 03	6.55*E* + 03
	STD	5.21*E* + 01	4.86*E* + 01	5.32*E* − 02	3.11*E* − 02	6.23*E* + 01	7.26*E* + 01

*f*_7_	AVG	1.12*E* + 04	1.36*E* + 04	7.53*E* + 01	8.30*E* + 03	1.26*E* + 02	1.44*E* + 04
	STD	7.55*E* + 02	3.86*E* + 02	9.37*E* + 01	2.63*E* + 00	7.17*E* + 02	6.71*E* + 02

*f*_8_	AVG	9.31*E* + 03	1.13*E* + 04	8.80*E* + 01	8.71*E* + 01	1.05*E* + 04	1.12*E* + 04
	STD	2.27*E* + 02	1.24*E* + 02	2.52*E* + 02	2.09*E* + 02	2.53*E* + 02	2.28*E* + 02

*f*_9_	AVG	4.03*E* + 04	9.02*E* + 04	1.04*E* + 04	1.08*E* + 04	8.87*E* + 02	9.18*E* + 04
	STD	8.67*E* + 01	1.26*E* + 02	3.21*E* + 03	2.31*E* + 01	1.02*E* + 04	2.14*E* + 04

*f*_10_	AVG	4.66*E* + 04	8.43*E* + 02	2.13*E* + 04	2.01*E* + 02	7.48*E* + 04	8.35*E* + 02
	STD	5.35*E* + 01	3.33*E* + 01	5.94*E* + 02	1.21*E* + 04	7.92*E* + 03	3.43*E* + 01

*f*_11_	AVG	1.23*E* + 04	6.92*E* + 04	1.23*E* + 04	1.24*E* + 04	3.16*E* + 04	3.17*E* + 04
	STD	2.87*E* + 02	1.71*E* + 01	1.55*E* + 03	6.34*E* + 02	1.12*E* + 04	5.68*E* + 03

*f*_12_	AVG	1.31*E* + 07	6.21*E* + 08	6.26*E* + 02	5.68*E* + 03	1.34*E* + 08	1.28*E* + 05
	STD	7.82*E* + 04	2.34*E* + 08	4.55*E* + 02	1.08*E* + 06	9.70*E* + 07	3.41*E* + 07

*f*_13_	AVG	1.61*E* + 03	3.36*E* + 08	9.85*E* + 04	1.59*E* + 05	2.58*E* + 09	3.81*E* + 07
	STD	1.13*E* + 03	1.52*E* + 08	9.45*E* + 02	1.66*E* + 06	2.04*E* + 07	1.35*E* + 07

*f*_14_	AVG	4.64*E* + 04	1.65*E* + 05	2.73*E* + 03	2.49*E* + 06	1.25*E* + 08	2.22*E* + 04
	STD	3.56*E* + 04	1.05*E* + 07	2.35*E* + 03	2.22*E* + 05	6.77*E* + 04	1.30*E* + 04

*f*_15_	AVG	5.32*E* + 04	1.02*E* + 07	4.97*E* + 04	1.49*E* + 05	4.41*E* + 07	6.90*E* + 09
	STD	4.33*E* + 04	9.34*E* + 06	4.60*E* + 04	2.10*E* + 03	4.11*E* + 05	3.08*E* + 08

*f*_16_	AVG	2.85*E* + 02	6.03*E* + 04	2.44*E* + 04	2.41*E* + 02	3.71*E* + 04	3.66*E* + 04
	STD	3.03*E* + 03	9.41*E* + 03	2.94*E* + 02	2.86*E* + 01	4.47*E* + 03	1.95*E* + 03

*f*_17_	AVG	2.43*E* + 02	4.11*E* + 04	1.95*E* + 02	1.97*E* + 02	2.55*E* + 04	2.65*E* + 04
	STD	2.76*E* + 01	1.03*E* + 02	9.28*E* + 02	1.39*E* + 03	2.53*E* + 03	1.15*E* + 03

*f*_18_	AVG	1.35*E* + 07	1.27*E* + 06	1.01*E* + 07	1.01*E* + 07	6.55*E* + 05	4.47*E* + 05
	STD	1.35*E* + 05	1.04*E* + 06	5.08*E* + 05	1.50*E* + 07	3.44*E* + 07	1.99*E* + 05

*f*_19_	AVG	8.86*E* + 04	9.87*E* + 06	1.81*E* + 03	1.81*E* + 03	6.41*E* + 05	1.13*E* + 07
	STD	6.38*E* + 04	9.43*E* + 06	6.26*E* + 04	1.42*E* + 05	3.01*E* + 07	4.85*E* + 06

*f*_20_	AVG	2.63*E* + 04	2.78*E* + 04	2.26*E* + 02	2.26*E* + 02	2.75*E* + 02	2.77*E* + 04
	STD	2.01*E* + 03	1.06*E* + 03	2.22*E* + 02	1.40*E* + 01	1.99*E* + 03	1.47*E* + 03

*f*_21_	AVG	2.43*E* + 04	2.38*E* + 04	2.36*E* + 04	2.38*E* + 04	2.62*E* + 02	2.58*E* + 04
	STD	2.52*E* + 02	6.12*E* + 02	5.14*E* + 03	3.33*E* + 02	3.87*E* + 02	1.68*E* + 02

*f*_22_	AVG	5.53*E* + 02	3.95*E* + 04	4.31*E* + 02	4.31*E* + 02	8.76*E* + 02	4.91*E* + 05
	STD	2.23*E* + 02	3.34*E* + 01	1.26*E* + 02	2.13*E* + 01	1.32*E* + 04	1.95*E* + 04

*f*_23_	AVG	3.14*E* + 04	3.47*E* + 04	2.73*E* + 04	2.73*E* + 02	3.22*E* + 04	2.96*E* + 02
	STD	7.76*E* + 02	2.33*E* + 01	1.44*E* + 03	2.61*E* + 02	8.91*E* + 02	2.72*E* + 02

**Table 5:** Classification rate (CR%) to adjust the population size of different algorithms for the data set is obtained from parts 2 and 3.

Algorithm	WOA		FWOA		ChOA		PGO		CVOA		BWO	
Population size	CR%	Time (s)	CR%	Time (s)	CR%	Time (s)	CR%	Time (s)	CR%	Time (s)	CR%	Time (s)
60	31.28	0.314	35.67	0.246	31.00	0.254	22.14	0.377	33.16	0.733	27.42	0.411
80	37.98	0.377	42.11	0.299	35.11	3.008	29.06	0.408	38.93	986.4	34.73	1.819
100	46.85	0.461	59.61	3.113	45.31	0.355	35.02	0.483	48.70	1.075	43.04	2.167
120	59.98	0.522	64.78	3.785	56.21	0.477	48.60	0.567	61.58	1.306	53.89	2.483
140	71.25	0.608	75.00	0.407	67.18	0.519	63.75	0.658	73.77	1.668	66.82	3.150
160	82.65	0.820	88.25	0.485	81.99	0.686	80.09	0.633	84.35	1.761	78.50	3.445
180	91.42	0.791	94.97	0.531	90.66	0.794	88.62	0.715	92.73	1.809	89.15	4.173
190	92.02	1.087	94.99	1.010	91.10	0.819	89.22	1.764	92.98	2.060	89.83	4.890
200	92.64	1.964	95.00	1.973	92.00	0.921	91.45	3.234	93.07	2.364	90.13	5.789

**Table 6:** The initial parameters and primary values of the benchmark algorithms.

Algorithm	Parameter	Value
WOA	*a*	Linearly decreased from 2 to 0
	Population size	180

FWOA	*a*	Tuning by fuzzy system
	Population size	180

ChOA	*a*	[1, 1]
	f	Linearly decreased from 2 to 0
	Population size	180

PGO	DR	0.3
	DRS	0.15
	EDR	0.8
	Population size	180

CVOA	P-die	0.05
	P_isolation	0.8
	P_superspreader	0.1
	P_reinfection	0.02
	Social_distancing	8
	P_travel	0.1
	Pandemic_duration	30
	Population size	180

BWO	PP	0.6
	CR	0.44
	PM	0.4
	Population size	180

**Table 7:** Results obtained from different algorithms for dataset reference [42].

Algorithm	MSE (AVE ± STD)	*p* values	A20 index	AUC (%)	Classification rate %
MLP-CVOA	0.1089 ± 0.1285	0.045	1	94.68	92.1283
MLP-WOA	0.1156 ± 0.1301	1.8534e-31	1	93.09	90.6422
MLP-FWOA	0.1002 ± 0.1154	0.027	1	97.71	93.0150
MLP-ChOA	0.1383 ± 0.1389	2.1464e-61	1	92.71	89.9476
MLP-PGO	0.1694 ± 0.1652	3.0081e-77	1	91.48	88.1115
MLP-BWO	0.1495 ± 0.1543	2.9813e-43	1	88.97	89.1014

**Table 8:** Results obtained from different algorithms for datasets obtained in parts 2 and 3.

Algorithm	MSE (AVE ± STD)	*p* values	A20 index	AUC (%)	Classification rate %
MLP-CVOA	0.0857 ± 0.1021	0.009	1	94.32	92.8027
MLP-WOA	0.0908 ± 0.1622	0.042	1	93.05	91.3455
MLP-FWOA	0.0689 ± 0.1031	0.013	1	95.89	94.9801
MLP-ChOA	0.1090 ± 0.1076	1.0589e-34	1	92.76	90.2410
MLP-PGO	0.1596 ± 0.1167	3.0013e-11	1	90.66	88.1004
MLP-BWO	0.1194 ± 0.1622	2.9854e-64	1	84.25	89.0478

## Data Availability

No data were used to support this study.
